# A Two-Axis Orthogonal Resonator for Variable Sensitivity Mode Localization Sensing

**DOI:** 10.3390/s24134038

**Published:** 2024-06-21

**Authors:** Yuta Nagasaka, Alessia Baronchelli, Shuji Tanaka, Takashiro Tsukamoto

**Affiliations:** 1 Department of Robotics, School of Engineering, Tohoku University, Sendai 980-8579, Japan; nagasaka.yuta.s5@dc.tohoku.ac.jp (Y.N.); mems@tohoku.ac.jp (S.T.); 2 Department of Civil and Environmental Engineering, Politecnico di Milano, 20133 Milano, Italy; alessia.baronchelli@mail.polimi.it

**Keywords:** MEMS resonator, mode localization, sensitivity tuning

## Abstract

This paper experimentally demonstrates a mode localization sensing approach using a single two-axis orthogonal resonator. The resonator consists of concentric multi-rings connected by elliptic springs that enable two orthogonal oscillation modes. By electrostatically tuning the anisotropic stiffness between the two axes, the effective coupling stiffness between the modes can be precisely controlled down to near-zero values. This allows the sensitivity of mode localization sensing to be tuned over a wide range. An order of magnitude enhancement in sensitivity is experimentally achieved by reducing the coupling stiffness towards zero. The resonator’s simple single-mass structure offers advantages over conventional coupled resonator designs for compact, tunable mode localization sensors. Both positive and negative values of coupling stiffness are demonstrated, enabling maximum sensitivity at the point where coupling crosses through zero. A method for decomposing overlapping resonance peaks is introduced to accurately measure the amplitude ratios of the localized modes even at high sensitivities. The electrostatic tuning approach provides a new option for realizing variable sensitivity mode localization devices using a simplified resonator geometry.

## 1. Introduction

Mode localization is the phenomenon that detects the small stiffness or mass perturbation applied to weakly coupled resonators [1,2,3,4,5]. Recently, MEMS sensors utilizing mode localization have gained attention for their potential to significantly enhance sensitivity and have been applied to various sensors such accelerometers [6,7,8,9,10], gyroscopes [11], electric current sensors [12], electrometers [13,14] magnetometers [15], and mass sensors [16,17,18]. One major advantage of mode localization sensing is that the change in amplitude ratio is much larger than that in resonant frequency [3]. Indeed, it has been demonstrated that mode localization sensors can achieve sensitivity approximately 1000 times higher than frequency-based sensors [4], enabling them to detect the same disturbances with higher sensitivity. Additionally, their high sensitivity allows them to reduce common noise sources such as temperature [19,20] and pressure [20,21].

Sensitivity is inversely proportional to coupling stiffness. However, attempts to increase sensitivity by reducing the coupling stiffness face challenges related to machining precision. Therefore, minimum coupling stiffness is usually limited by fabrication precision. To breakthrough this limit, a lot of methods to reduce the effective coupling between resonators have been proposed. Thiruvenkatanathan and Seshia used electrostatic coupling instead of mechanical coupling [4]. However, electrostatic coupling may bring stiffness instability. Humbert et al. proposed external electric coupling [22]. However, the coupling effect was not small, resulting in low sensitivity. Kang et al. reported that a center-anchored three degrees-of-freedom (DOF) mass resonator reduces the effective coupling stiffness and improves the sensitivity by 349% [7]. The same research group reported a four DOF resonator to further improve the sensitivity [23]. Chen et al. reported a three DOF resonator in which effective coupling stiffness could be controlled by the stiffness balance between springs [24,25]. Matthew et al. reported a 15 degree-of-freedom system using connected cantilevers [26]. Increasing the degrees of freedom of resonators in sensors indeed leads to a significant improvement in sensitivity. However, this also increases the number of resonators required, leading to larger structural footprints. Zhou et al. reported that the two-axis resonator showed mode localization [27] and mentioned the possibility of stiffness tuning. However, sensitivity tuning was not experimentally demonstrated. In this study, we experimentally demonstrated mode localization sensing with tunable sensitivity using a two-axis orthogonal resonator.

## 2. Working Principle

### 2.1. Two-Axis Orthogonal Resonator

**Figure 1:**
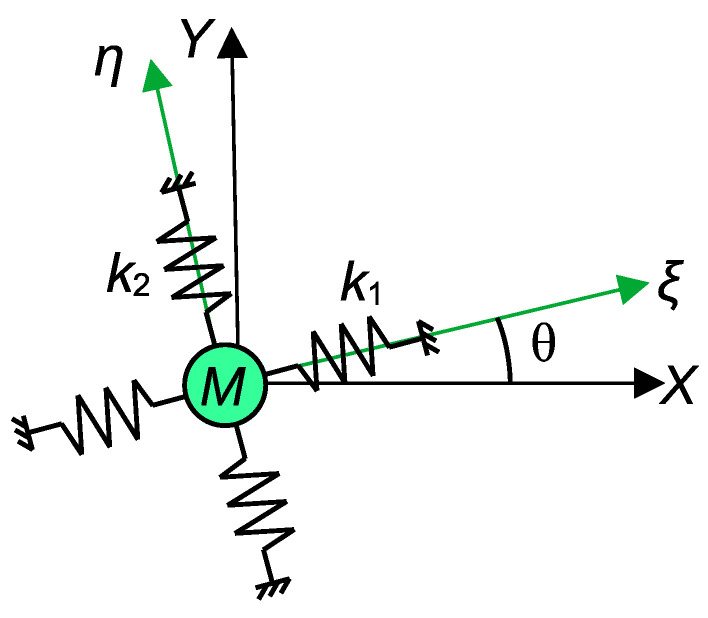
Two-axis resonator model.

Figure 1 shows the simplified single-mass, two-axis orthogonal resonator. *M* is a mass, and $k_{1}$ and $k_{2}$ are the pairs of springs in two orthogonal directions. Please note that the stiffness indicated in the figure represents the total stiffness, which includes not only the mechanical stiffness arising from the MEMS spring structure but also the stiffness variation due to electrostatic forces. In general, the direction of the springs (i.e., the principal axes of stiffness) are not aligned with the horizontal (X) and vertical (Y) axes. Let θ be the angle between the principal axis and X-Y axes. The stiffness matrix in the ξ−η coordinate can be expressed as follows:

(1) $$\begin{array}{c} K_{0}=\left[\begin{array}{cc} k_{1} & 0\\ 0 & k_{2} \end{array}\right]. \end{array}$$

The stiffness in the X-Y coordinate system can be obtained using the coordinate transformation matrix, E(θ), as follows:

(2) $$\begin{array}{c} K=E\left(\theta \right)K_{0}E^{-1}\left(\theta \right)=\left[\begin{array}{cc} k_{1}\cos^{2}\left(\theta \right)+k_{2}\sin^{2}\left(\theta \right) & \left(k_{1}-k_{2}\right)\sin\left(\theta \right)\cos\left(\theta \right)\\ \left(k_{1}-k_{2}\right)\sin\left(\theta \right)\cos\left(\theta \right) & k_{1}\sin^{2}\left(\theta \right)+k_{2}\cos^{2}\left(\theta \right) \end{array}\right]\equiv \left[\begin{array}{cc} k_{x} & -k_{c}\\ -k_{c} & k_{y} \end{array}\right], \end{array}$$

where

(3) $$\begin{array}{c} E\left(\theta \right)=\left[\begin{array}{cc} \cos(\theta ) & -\sin(\theta )\\ \sin(\theta ) & \cos(\theta ) \end{array}\right]. \end{array}$$

This expression resembles the stiffness matrix of a “normal” mode-localized resonator,

(4) $$\begin{array}{c} K^{\prime }=\left[\begin{array}{cc} k_{x}^{\prime }+k_{c}^{\prime } & -k_{c}^{\prime }\\ -k_{c}^{\prime } & k_{y}^{\prime }+k_{c}^{\prime } \end{array}\right]. \end{array}$$

In many cases, the coupling stiffness of the resonators used in mode localized sensing, $k_{c}$, is small compared to the main stiffnesses, $k_{x}$ and $k_{y}$, and the coupling stiffness of the two-axis resonator can be expressed as follows:

(5) $$\begin{array}{c} k_{c}=\left(k_{2}-k_{1}\right)\sin\left(\theta \right)\cos\left(\theta \right). \end{array}$$

The stiffness $k_{1}$, $k_{2}$ as well as principle axis θ could be modified by electrostatic tuning; therefore, $k_{c}$ could be controlled down to zero.

**Figure 2:**
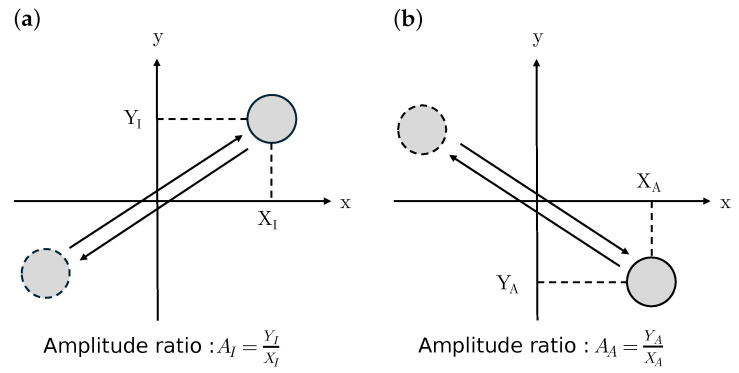
Schematic of modal shapes of the two-axis resonator. (**a**) In-phase (IP) and (**b**) anti-phase (AP) modes.

Figure 2 illustrates the two oscillation modes used in this paper: in-phase (IP) and anti-phase (AP) modes. For conventional mode-localized sensors, it is common to define two coordinates in parallel. In such a definition, in the IP mode, the two masses vibrate in the same direction, while in the AP mode, they vibrate in opposite directions. However, in our device, the two axes are arranged perpendicularly. Therefore, in the IP mode, the X-axis and Y-axis vibrate in the same direction (i.e., 45° direction), while in the AP mode, the X-axis and Y-axis vibrate in opposite directions (i.e., −45° direction). The resonant frequencies are solved as follows:

(6) $$\begin{array}{cc} \omega_{1} & =\sqrt{\frac{k_{0}+\frac{\Delta k}{2}-\sqrt{\frac{\Delta k^{2}}{4}+k_{c}^{2}}}{M}} \end{array}$$

(7) $$\begin{array}{cc} \omega_{2} & =\sqrt{\frac{k_{0}+\frac{\Delta k}{2}+\sqrt{\frac{\Delta k^{2}}{4}+k_{c}^{2}}}{M}}, \end{array}$$

where $k_{0}=\frac{k_{x}+k_{y}}{2}$ and $\Delta k=k_{y}-k_{x}$. The frequency difference takes the minimum value of

(8) $$\begin{array}{c} |\omega_{1}-\omega_{2}|∼\frac{|k_{c}|}{\sqrt{Mk_{0}}} \end{array}$$

under the balanced condition, such that Δk=0. The modal shapes are represented as the amplitude ratio, Y/X, which could be solved as follows:

(9) $$\begin{array}{cc} A_{1} & =\frac{-\Delta k+\sqrt{\Delta k^{2}+4k_{c}^{2}}}{2k_{c}} \end{array}$$

(10) $$\begin{array}{cc} A_{2} & =\frac{-\Delta k-\sqrt{\Delta k^{2}+4k_{c}^{2}}}{2k_{c}}. \end{array}$$

When the coupling stiffness is positive, $k_{c}>0$, modes 1 and 2 correspond to the IP and AP mode, respectively. On the other hand, when $k_{c}<0$, modes 1 and 2 correspond to the AP and IP mode, respectively. The sensitivity can be thus expressed as follows:

(11) $$\begin{array}{c} \frac{\partial A}{\partial (\Delta k)}=\left\{\begin{array}{cc} -\frac{1}{2k_{c}} & \left(\Delta k\ll k_{c}\right)\\ -\frac{1}{k_{c}} & (\Delta k\gg k_{c}). \end{array}\right. \end{array}$$

Therefore, the sensitivity could be enlarged by reducing the coupling stiffness, $k_{c}$.

### 2.2. Resonator Structure

**Figure 3:**
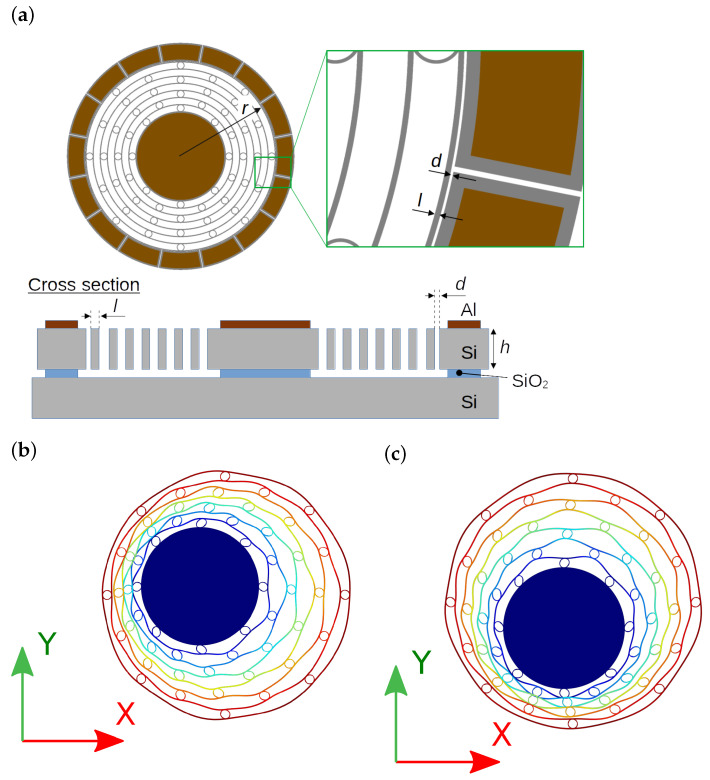
(**a**) Structure of the resonator and definition of the (**b**) X and (**c**) Y axes modal shapes.

Figure 3 shows the structure of the resonator and the definition of the axes used in this paper. The resonator consists of multiple concentric rings connected by elliptic springs [28]. However, in this paper, two n=1 modes, which correspond to the ring moving as a whole either horizontally or vertically, as shown in Figure 3b,c, are used. Sixteen electrostatic transducers are placed at the periphery of the ring, which are used for driving, sensing, and stiffness tuning. Representative dimensions of the multi-ring resonator are summarized in Table 1. The radius refers to the distance from the center of the resonator to the outermost ring. The width represents the thickness per ring, while the small gap denotes the distance between the outermost ring and the fixed electrode. The height indicates the device’s thickness. The multi-ring structure is connected to the substrate via a cylindrical anchor structure at the center.

The oscillation modes are estimated by the finite element method (FEM) using COMSOL Multiphysics Ver. 5.2. The resonant frequencies of the X (Figure 3b) and Y (Figure 3c) axes are 49.2 kHz and 49.3 kHz, respectively. The structure is symmetric; therefore, the difference between the two modes is considered a numerical error.

**Figure 4:**
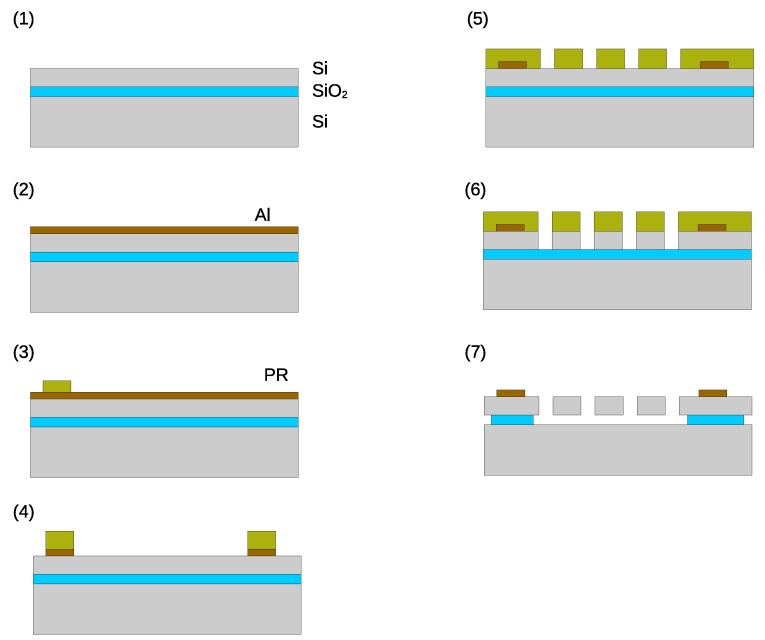
Fabrication process. (**1**) SOI wafer. (**2**) Al deposition (wet chemical etching). (**3**) Photoresist patterning. (**4**) Al etching. (**5**) Photoresist patterning. (**6**) Si etching (DRIE). (**7**) SiO_2_ etching (vapor phase HF).

**Figure 5:**
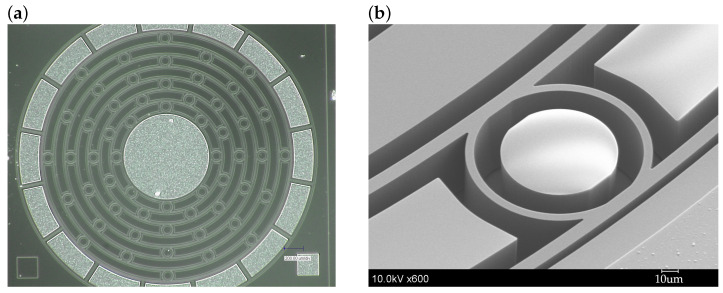
(**a**) Microscopic image and (**b**) scanning electron micrograph of the fabricated device.

The device is fabricated using the SOI process. Figure 4 shows the fabrication process. The thickness of the handle, buried oxide, and device layers are 400, 3, and 50 µm, respectively. The crystal orientation and resistivity of the device layer are (100) and 0.1 Ω·cm, respectively. An Al layer is deposited on the device layer. Then, the photoresist is deposited and patterned. The thin Al film is patterned by wet chemical etching using a photoresist as the etching mask. The photoresist is removed and the thin Al film is sintered for good electrical contact. Then, another layer of photoresist is deposited and patterned. The Si device layer is patterned by deep reactive ion etching (DRIE). After DIRE, the photoresist is removed and the wafer is diced into each chip. Finally, the buried oxide layer is removed by vapor-phase HF. Figure 5 shows the fabricated device.

### 2.3. Electrostatic Tuning

**Figure 6:**
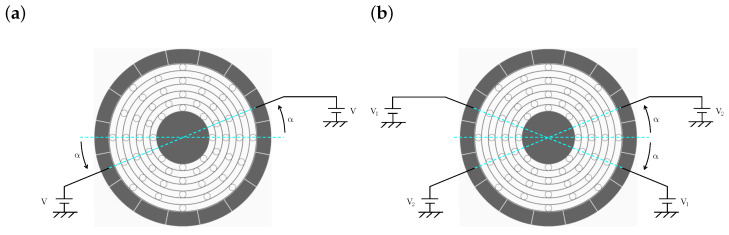
Electrode usage for electrostatic tuning. (**a**) Electrode pair direction α modifies the stiffness of this direction. (**b**) Tuning direction could be arbitrarily controlled by combining two sets of electrodes with two independent voltages, $V_{1}$ and $V_{2}$.

The bias voltage between the resonator and static electrode generates the virtual spring as follows:

(12) $$\begin{array}{c} K_{e,0}=-\epsilon_{0}\frac{S}{d^{3}}V^{2}, \end{array}$$

where $\epsilon_{0}$, *S*, *d*, and *V* are the permeability of the vacuum, the area of the electrode, the gap between moving and static electrodes, and the applied voltage, respectively. To generate the symmetric electrostatic force, a pair of electrodes, as shown in Figure 6, is used for tuning. The stiffness matrix change caused by the electrostatic tuning written in X–Y coordinate can be obtained by coordinate transformation as follows:

(13) $$\begin{array}{c} K_{e}\left(\alpha ,V\right)=2E\left(\alpha \right)\left[\begin{array}{cc} K_{e,0} & 0\\ 0 & 0 \end{array}\right]E^{-1}\left(\alpha \right) \end{array}$$

(14) $$\begin{array}{cc} \; & =-2\epsilon_{0}\frac{S}{d^{3}}V^{2}\left[\begin{array}{cc} \cos^{2}\left(\alpha \right) & \sin(\alpha )\cos(\alpha )\\ \sin(\alpha )\cos(\alpha ) & \sin^{2}\left(\alpha \right) \end{array}\right], \end{array}$$

where α is the direction of the electrode pair. A factor of 2 in the equation originates from the fact that there are two paired electrodes, which doubles the tuning range. When using two pairs of electrodes with directions of α and −α, the stiffness modification factor is as follows:

(15) $$\begin{array}{cc} \Delta K & =K_{e}\left(\alpha ,V_{2}\right)+K_{e}\left(-\alpha ,V_{1}\right)\\ & =-2\epsilon_{0}\frac{S}{d^{3}}\left[\begin{array}{cc} \left(V_{2}^{2}+V_{1}^{2}\right)\cos^{2}\left(\alpha \right) & \left(V_{2}^{2}-V_{1}^{2}\right)\sin\left(\alpha \right)\cos\left(\alpha \right)\\ \left(V_{2}^{2}-V_{1}^{2}\right)\sin\left(\alpha \right)\cos\left(\alpha \right) & \left(V_{2}^{2}+V_{1}^{2}\right)\sin^{2}\left(\alpha \right) \end{array}\right], \end{array}$$

where $V_{1}$ and $V_{2}$ are the applied voltages to the pair of electrodes, as shown in Figure 6b. The diagonal and non-diagonal terms control the X–Y stiffness mismatch and coupling stiffness, respectively.

### 2.4. Experimental Setup

**Figure 7:**
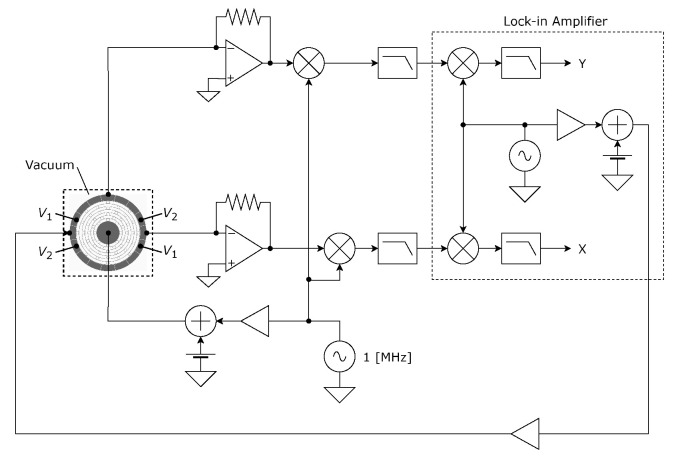
Experimental setup.

**Figure 8:**
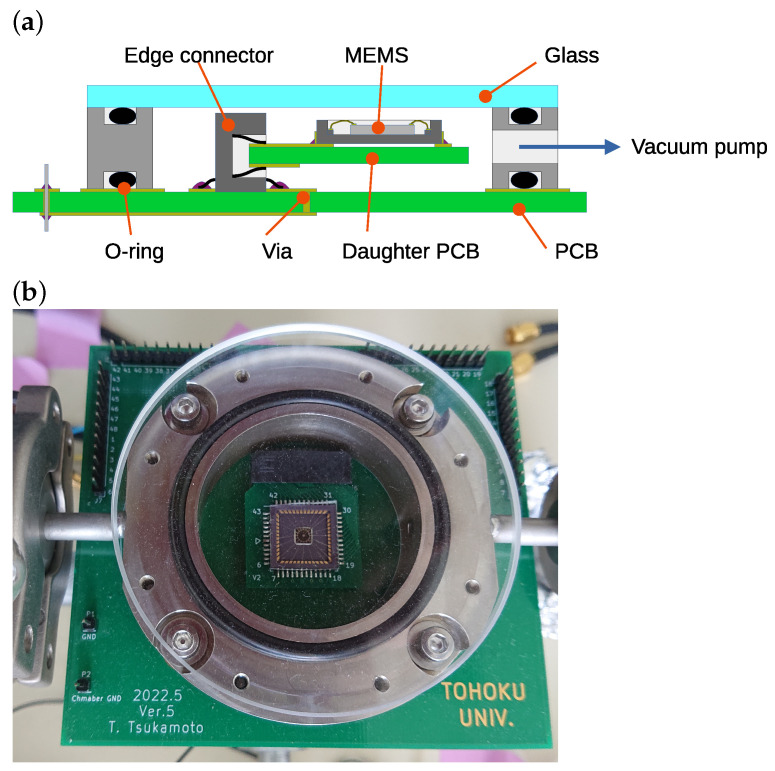
Vacuum chamber with electrical feed-through made of PCB. (**a**) Schematic cross-sectional view and (**b**) photograph.

Figure 7 shows the experimental setup. Since the resonator is not vacuum packaged, it is placed in vacuum equipment consisting of a metal chamber, a glass top plate, and a bottom plate with electrical feed-throughs made by printed circuit board (PCB). Figure 8 shows the schematic and photograph of the developed vacuum chamber. The leak rate is estimated as small as $1\times 10^{-8}\;\mathrm{Pa}\cdot m^{3}/s$. A pressure level as low as $10^{-2}$ Pa could be observed using the diffusion pump. However, only the rotary pump is used for the resonance measurement. Thus, the inside pressure during resonance measurement is approximately 0.6 Pa. Oscillation displacement is detected by the capacitance of sensing electrodes. A high-frequency sinusoidal signal and DC bias are applied to the resonator. The modulation frequency, amplitude, and bias voltages are 1 MHz, 1 V_pk_, and 4 V, respectively. The displacement is obtained by the synchronous demodulation technique. The amplitudes and phases of oscillation are detected using a lock-in amplifier (UHF2LI, Zurich Instruments Ltd., Zurich, Switzerland). The actuation signal is generated by the lock-in amplifier and applied to the X-axis driving electrode through a voltage amplifier. Tuning voltages, $V_{1}$ and $V_{2}$, are applied by regulated power sources (P4K-80M, Matsusada Precision, Inc., Kusatsu, Japan).

## 3. Experimental Results

### 3.1. Frequency Response

#### 3.1.1. As-Fabricated Condition

**Figure 9:**
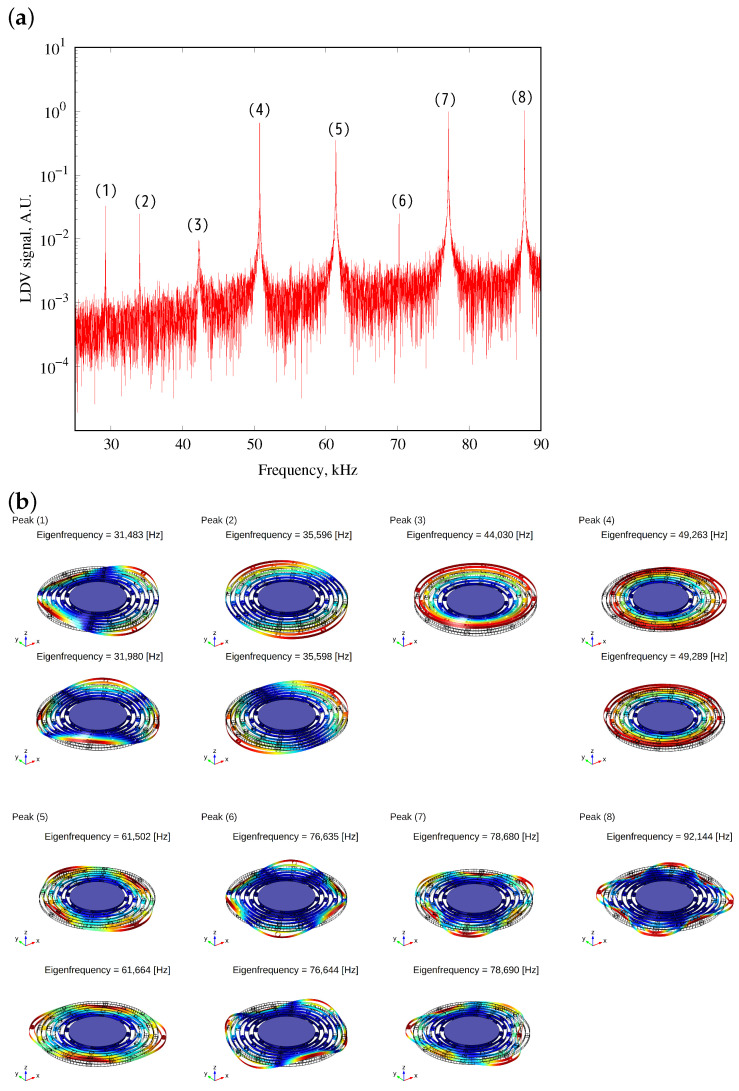
(**a**) Frequency response of device detected by LDV and (**b**) corresponding modal shapes obtained by FEA. In this study, oscillation modes at peak (4) were used for the following experiment.

First of all, the resonance of the fabricated device was measured. To avoid the electrostatic tuning effect caused by the applied voltages, the device was actuated by the external piezoelectric actuator and the displacement was detected by a laser Doppler vibrometer (LDV). The piezoelectric actuator induces out-of-plane vibrations in the device. However, due to the fact that the device and the actuator are not perfectly orthogonal and the device possesses a very high Q-factor, in-plane vibration modes can also be excited. Additionally, the LDV detects in-plane vibrations by measuring from a direction that is tilted 24° from the vertical. Figure 9 shows the obtained resonant peaks from 25 kHz to 90 kHz. Due to the symmetric structure, some peaks are degenerated. The modal shapes of oscillation modes predicted by the FEM are shown in Figure 9. Two oscillation modes at the position of peak-(4) were used in the following experiments.

**Figure 10:**
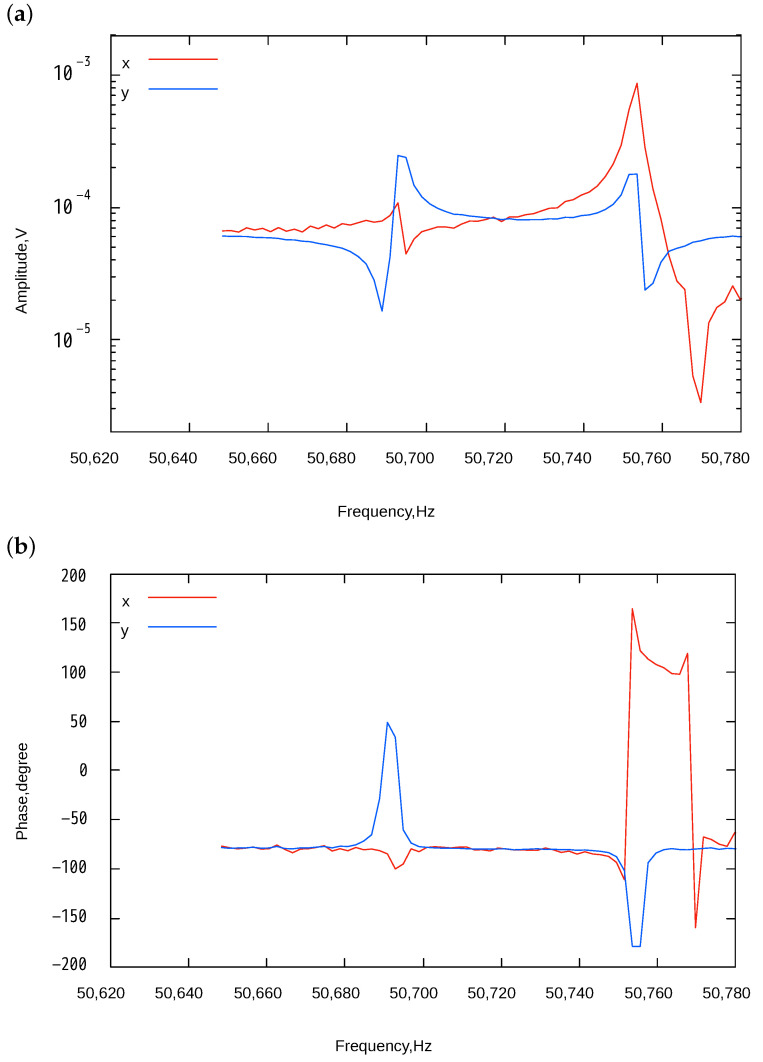
Frequency response of the two-axis resonator without applying voltages to $V_{1}$ and $V_{2}$. (**a**) Amplitudes and (**b**) phases of the X and Y axes.

Figure 10 shows the frequency response when no tuning voltage was applied to $V_{1}$ and $V_{2}$. Two resonant peaks, corresponding to AP and IP modes, were observed. From the detected phase differences, the first and second peaks correspond to AP and IP modes, respectively. Negative peaks shown in the figure come from the drive-to-sens feed-through signal. At the specific frequency, signals generated by MEMS motion and feed-through have the same amplitude and opposite phase, cancelling each other out. When the feed-through signal is much larger than that from MEMS motion, the detected phase is mainly dominated by the feed-through signal. Thus, at frequencies far from the resonance point, the phase is influenced by the feed-through signal, resulting in a constant phase in these regions. Conversely, as the frequency approaches the resonance point, the MEMS vibration signal increases sharply, and the observed phase shifts to that of the resonance signal. Therefore, a rapid phase change is observed around the resonance point. The as-fabricated frequency mismatch was approximately 60 Hz.

#### 3.1.2. Effect of Electrostatic Tuning

**Figure 11:**
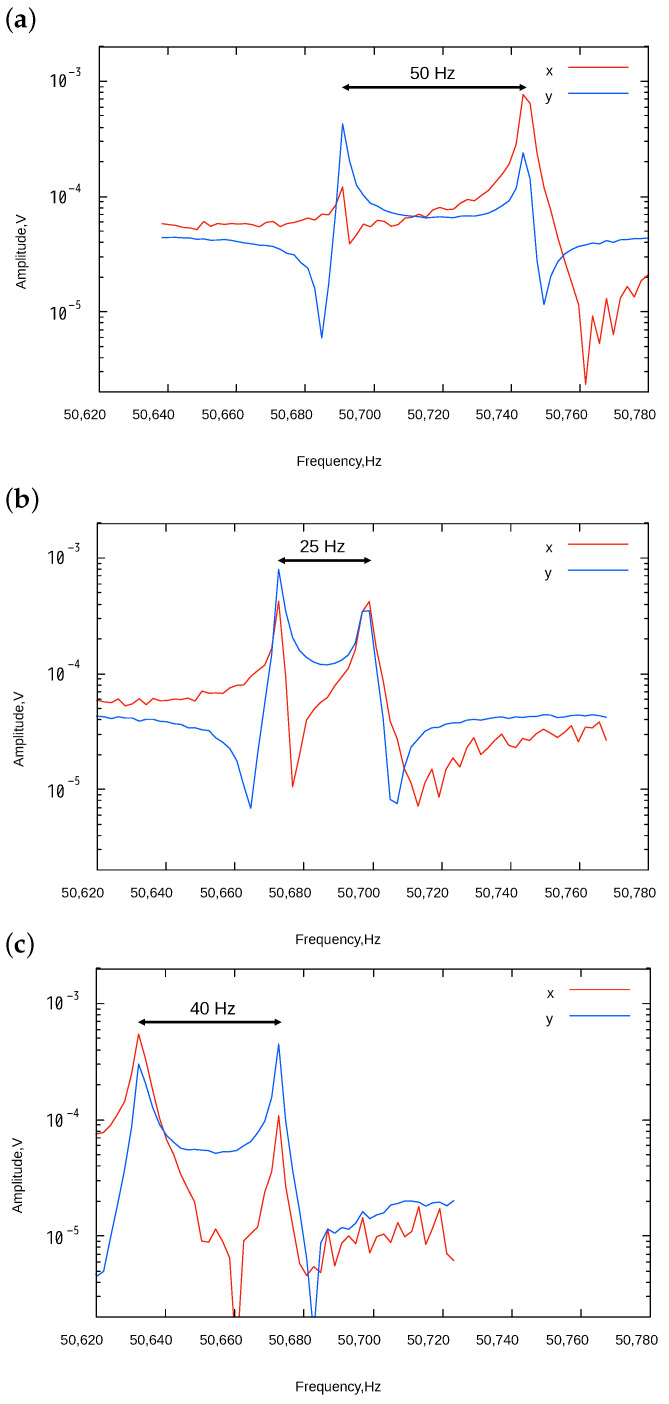
X and Y axes frequency response under different electrostatic tunings. $V_{1}$ and $V_{2}$ were applied under the condition that $V_{1}=V_{2}=$ (**a**) 20 V, (**b**) 45 V, and (**c**) 60 V.

First, only orthogonal terms, i.e., stiffness mismatch between X and Y axes, were controlled by applying tuning under the condition that $V_{1}=V_{2}\equiv V_{T}$. From Equation (15), only orthogonal terms could be modified. Figure 11 shows the measured frequency response under the tuning conditions of $V_{T}=20$, 45, and 60 V. When $V_{T}=20$ V, the frequency mismatch became smaller compared to the as-fabricated condition (Figure 10). The mismatch became the minimum around $V_{T}=45$ V, and the amplitude ratio of both modes approached unity. The minimum frequency mismatch was approximately 30 Hz. This indicates that the stiffness mismatch was minimized in this condition. The mismatch increased when the tuning voltage became V=60 V.

**Figure 12:**
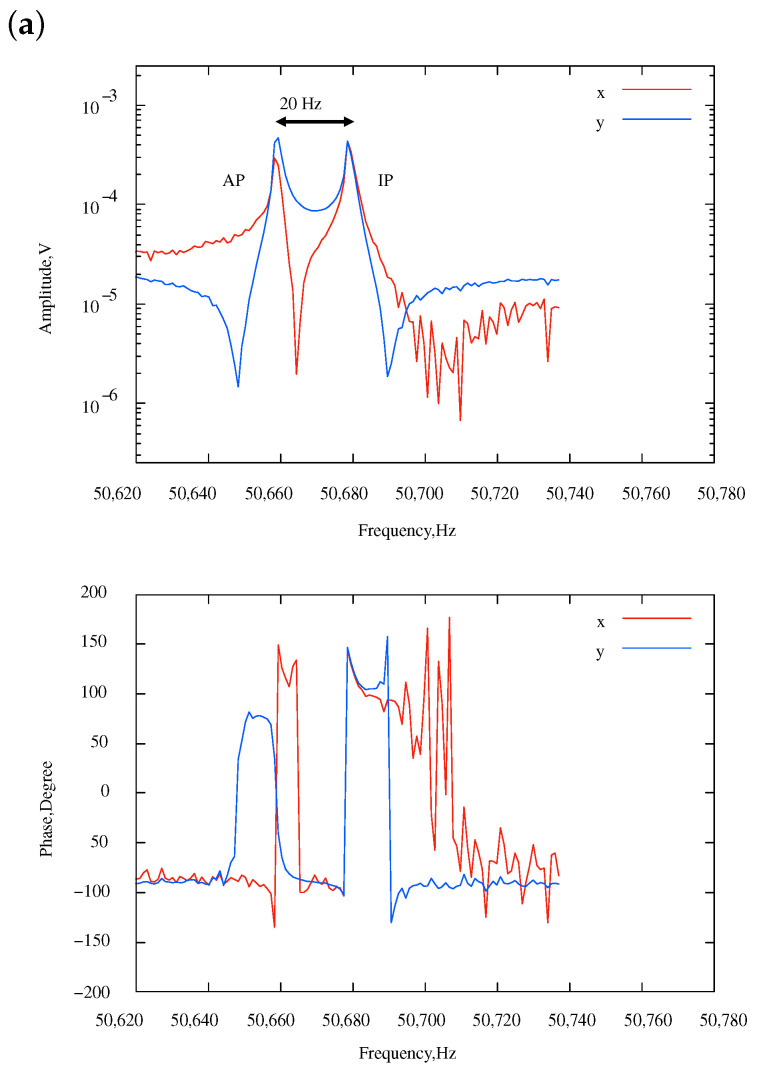

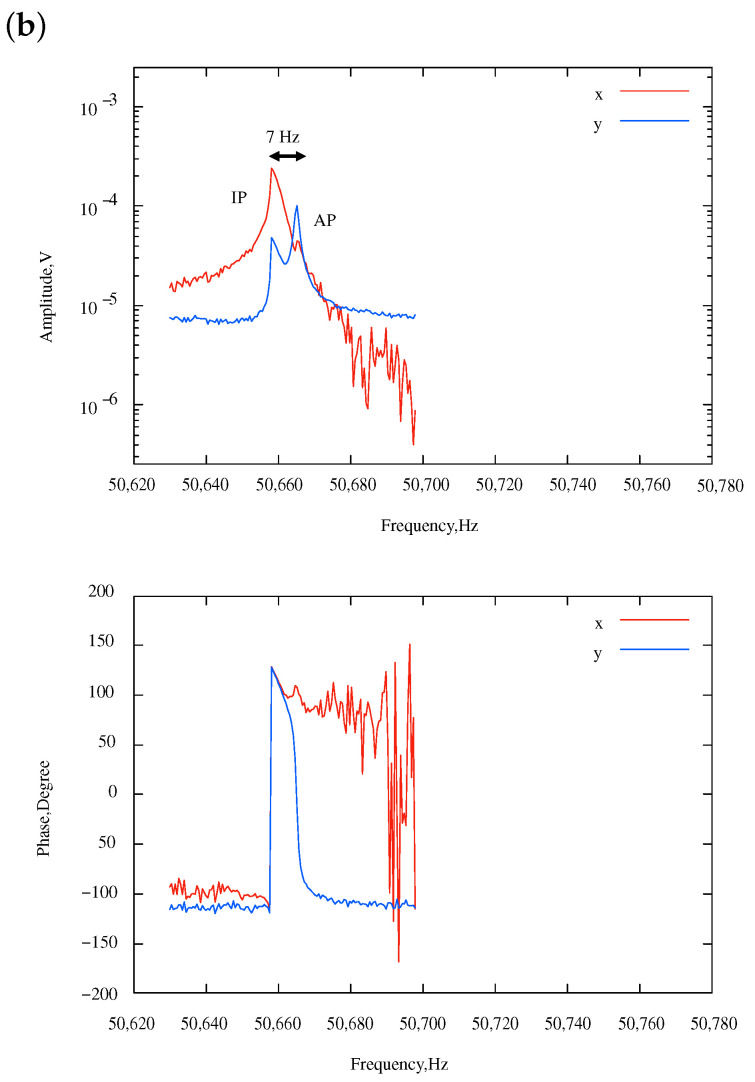
Amplitude and phase frequency response under unbalanced electrostatic tunings (i.e., $V_{1}\neq V_{2}$). $\{V_{1},V_{2}\}=$ (**a**) {45 V, 50 V} and (**b**) {40 V, 60 V}.

Then, the non-diagonal term, i.e., the coupling stiffness, was controlled by an unbalanced tuning condition, $V_{1}\neq V_{2}$. According to Equation (15), the coupling stiffness and the stiffness unbalance were controlled by $V_{2}^{2}-V_{1}^{2}$ and $V_{1}^{2}+V_{2}^{2}$, respectively. Figure 12 shows some examples of the experimental results. The minimum frequency mismatch was reduced to approximately 20 Hz under the tuning condition that $\left(V_{1},\;V_{2}\right)=\left(45\;V,\;50\;V\right)$, at which the effective coupling stiffness became small (Equation (8)). In this condition, the frequency of the AP mode was smaller than that of the IP mode, indicating that $k_{c}$ was negative. The minimum frequency mismatch was further reduced to 7 Hz when $\left(V_{1},\;V_{2}\right)=\left(40\;V,\;60\;V\right)$. In this condition, the frequency order was swapped, which means $k_{c}$ became positive. Theoretically, $k_{c}$ becomes zero in between these two conditions, which means the sensitivity becomes infinity.

As $k_{c}$ approaches zero, the sensitivity increases. Therefore, the amplitude ratios deviate significantly from 1 even under the small stiffness perturbation. This implies that the direction of the eigenmodes (eigenvectors) approaches the X or Y axes. For example, the AP mode in Figure 12b had an eigenmode that is nearly aligned with the Y axis. Due to this, under the X-axis excitation conducted in this experiment, efficient excitation could not be achieved, resulting in reduced amplitude.

### 3.2. Method for Amplitude Ratio Detection

**Figure 13:**
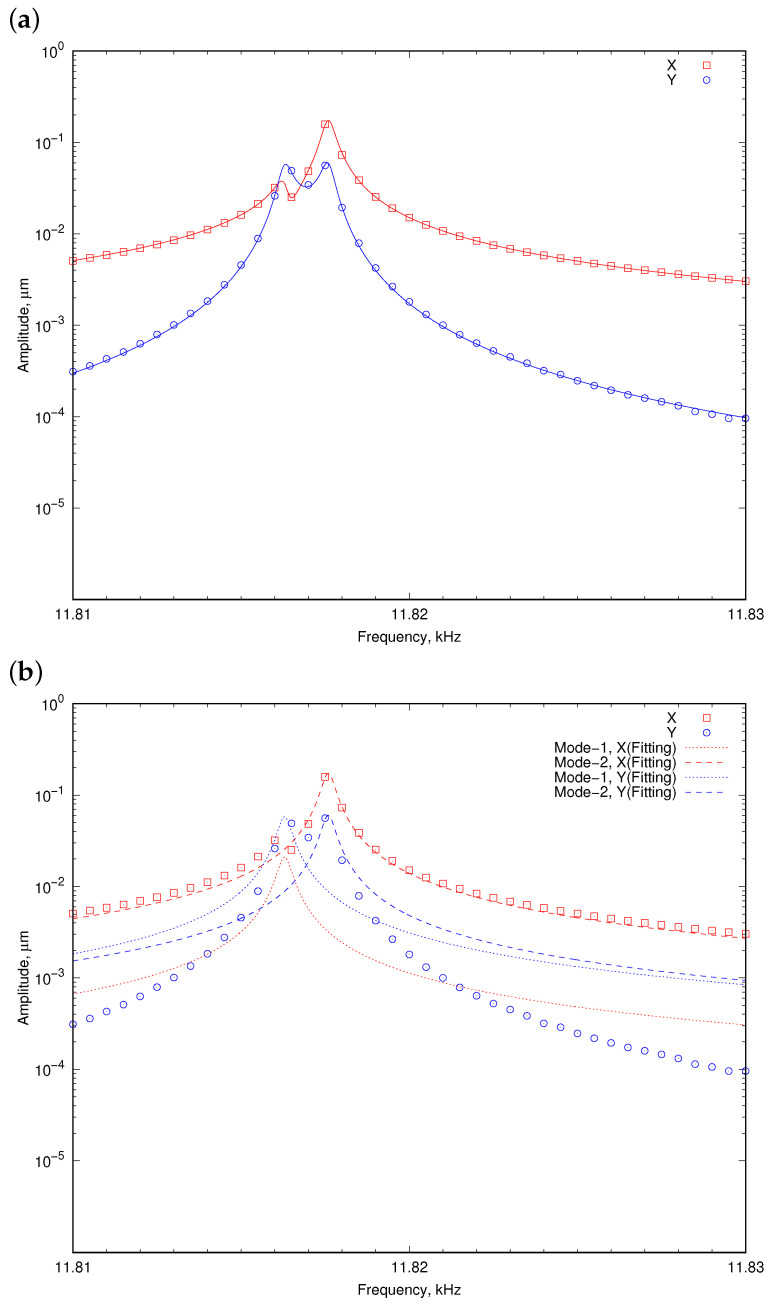
Fitting result using double Lorentz function fitting. (**a**) Overall and (**b**) decomposed into each components.

When two oscillation modes approach in the frequency domain, two resonances partly overlap, introducing difficulty in measuring the amplitude ratio. This issue becomes particularly evident with high-sensitivity sensors. Therefore, it is necessary to develop a method for detecting the amplitude ratio of each peak even when two peaks overlap. Because two modes have different phases, two Lorentz functions are overlaid in the complex space as follows:

(16) $$\begin{array}{c} \left\{\begin{array}{cc} X & =A_{X,1}\frac{\omega_{1}^{2}/Q_{1}}{\left(\omega_{1}^{2}-\omega^{2}\right)+i\omega_{1}\omega /Q_{1}}+A_{X,2}\frac{\omega_{2}^{2}/Q_{2}}{\left(\omega_{2}^{2}-\omega^{2}\right)+i\omega_{2}\omega /Q_{2}}\\ Y & =A_{Y,1}\frac{\omega_{1}^{2}/Q_{1}}{\left(\omega_{1}^{2}-\omega^{2}\right)+i\omega_{1}\omega /Q_{1}}+A_{Y,2}\frac{\omega_{2}^{2}/Q_{2}}{\left(\omega_{2}^{2}-\omega^{2}\right)+i\omega_{2}\omega /Q_{2}}, \end{array}\right. \end{array}$$

where $A_{X,1}$, $A_{X,2}$, $A_{Y,1}$ and $A_{Y,2}$ are the X- and Y-axis amplitudes of mode 1 and 2, respectively. $\omega_{1}$, $\omega_{2}$, $Q_{1}$ and $Q_{2}$ are the resonant frequencies and Q-factors of mode-1 and 2, respectively. Those parameters were used for fitting. Figure 13 shows a example of the fitting. Please note that the data shown in Figure 13 are not from the resonator shown in Figure 3; therefore, the resonant frequency was different. However, the proposed method does not depend on the structures and oscillation modes of the resonator. Thus, the method could be applicable. As can be seen, two resonant peaks partly overlap, and it is difficult to directly measure the amplitude ratio of each peak. However, the proposed function fits to the experimental results well (Figure 13a). In addition, the amplitudes could be decomposed into two resonant peaks, as shown in Figure 13b. From the decomposed peak amplitudes (corresponding to $A_{X,1}$, $A_{X,2}$, $A_{Y,1}$, and $A_{Y,2}$ in Equation (16)), the amplitude ratios of two resonant peaks could be obtained.

### 3.3. Mode Localization Measurement

The mode localization phenomenon was evaluated to apply the stiffness perturbation but keep the coupling stiffness at the constant value. Both stiffness perturbation and $k_{c}$ tuning were generated by electrostatic tuning. Considering Equation (15), stiffness perturbation could be controlled by $V_{2}^{2}+V_{1}^{2}$, while $k_{c}$ could be controlled by $V_{2}^{2}-V_{1}^{2}$. Thus, both parameters could be independently controlled. One set of measurements was carried out under a constant value of $V_{2}^{2}-V_{1}^{2}$, i.e., constant $k_{c}$, while changing $V_{2}^{2}+V_{1}^{2}$ to sweep the stiffness perturbation. Then, the value of $V_{2}^{2}-V_{1}^{2}$ was changed and another set of measurements were conducted. The stiffness perturbation is calculated as follows:

(17) $$\begin{array}{c} \Delta k=2\epsilon_{0}\frac{S}{d^{3}}\left(V_{1}^{2}+V_{2}^{2}\right)\left(\cos^{2}\left(\alpha \right)-\sin^{2}\left(\alpha \right)\right). \end{array}$$

**Figure 14:**
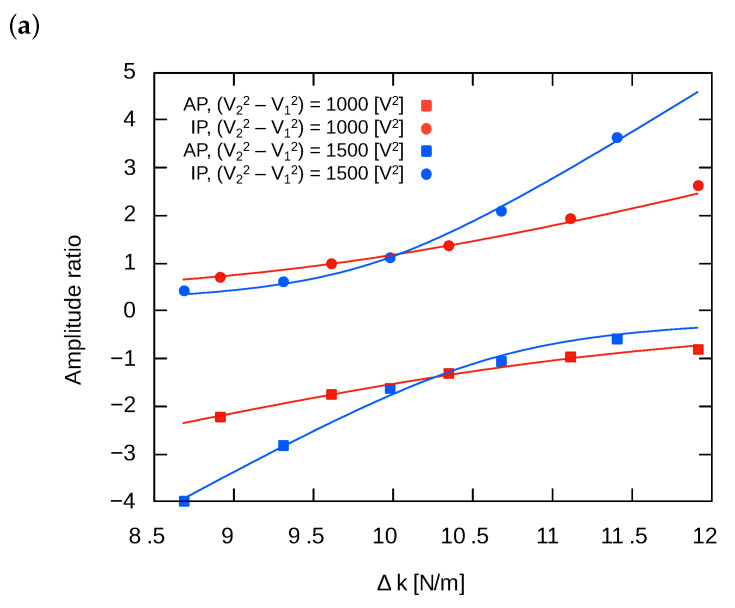

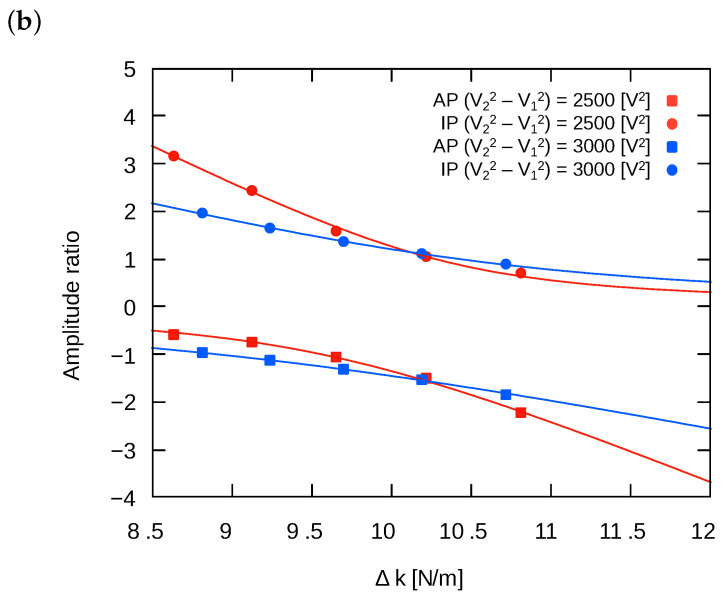
Amplitude ratios of IP and AP modes with (**a**) negative and (**b**) positive effective coupling stiffness.

**Figure 15:**
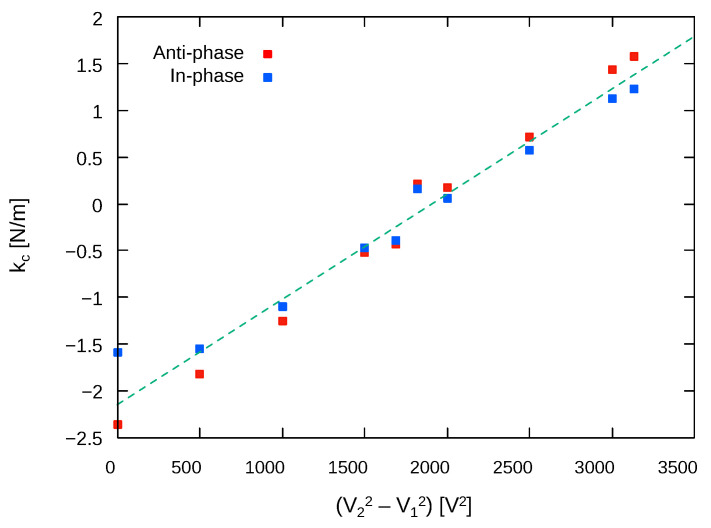
Tuning result of coupling stiffness.

The amplitude ratios were obtained by fitting the frequency response data to the double Lorentz functions, as described in Section 3.2. Figure 14 shows the obtained amplitude ratios. The data were fitted to the equation of the amplitude ratio (Equation (10)) using $k_{c}$ as the fitting parameter. The point near the amplitude ratio is 1 or −1, and the plots of experimental results with different $k_{c}$ intersect. This indicates that, at this point, the structural mismatch is perfectly compensated by electrostatic tuning, resulting in a state where $k_{x}=k_{y}$. From this result, it can be inferred that for the oscillator used in this experiment, the mismatch in the X and Y axes due to fabrication imperfection was approximately 10.2 N/m. Table 2 and Figure 15 summarize the obtained effective coupling stiffness. As can be seen, the amplitude ratio followed the theory well, and the effective stiffness could be controlled by the tuning voltages. The coupling stiffness linearly changed with respect to $V_{2}^{2}-V_{1}^{2}$, which means the proposed tuning method worked well. The tuning efficiency of effective coupling stiffness for AP and IP modes were $13\times 10^{-4}\;\left(N/m\right)/V^{2}$ and $9.8\times 10^{-4}\;\left(N/m\right)/V^{2}$, respectively. Those values are close to the theoretical value:

(18) $$\begin{array}{c} \frac{\partial k_{c}}{\partial (V_{2}^{2}-V_{1}^{2})}=2\epsilon_{0}\frac{S}{d^{3}}\sin\left(\alpha \right)\cos\left(\alpha \right)=11\times 10^{-4}\;\left[\left(N/m\right)/V^{2}\right]. \end{array}$$

Various types of oscillators for mode-localized sensors have been proposed. Among these, the method of varying coupling stiffness using electrostatic attraction between two oscillators is particularly effective [29,30,31], as it achieves variable sensitivity similar to our study. However, a notable distinction of this method is that the coupling stiffness is always negative and cannot change in sign. Additionally, another proposed method involves altering the effective coupling stiffness by adding oscillatory systems, which could also change the sensitivity in mode-localized sensors [24,25,31,32]. However, those resonators require additional components which make the whole system complex. These previous methods and the one proposed in this paper share similar objectives and achieved effects. Therefore, the major contribution of our research is providing a new option for variable mode-localized sensing. Furthermore, a significant advantage of our method is the use of a single mass, which simplifies the device structure and facilitates miniaturization compared to other approaches. Although similar methods to the one proposed in this study have been mentioned in the past [27], direct experimental validation has not been reported. The ability to experimentally measure variable sensitivity directly in this study is of significant importance.

## 4. Conclusions

In this paper, mode localization sensing using a single-mass, dual-axis resonator was experimentally demonstrated. We demonstrated that the coupling stiffness, and therefore, the sensitivity of mode localized sensing, can be electrostatically controlled by electrodes around the oscillator. Sensitivity tuning could be enhanced at least 10 times using the proposed method. Negative coupling stiffness was observed, which means coupling stiffness close to zero, i.e., high sensitivity, could be possible.

## Figures and Tables

**Table 1:** Parameters of the ring resonator.

Parameter	Character	Numerical Value
Radius	*r* [m]	$1.2\times 10^{-3}$
Width	*l* [m]	$1.0\times 10^{-5}$
Gap	*d* [m]	$5.0\times 10^{-6}$
Height	*h* [m]	$5\times 10^{-5}$

**Table 2:** Obtained effective coupling stiffness.

$V_{2}^{2}-V_{1}^{2}$ [V^2^]	kc [N/m] (Anti-Phase)	kc [N/m] (In-Phase)
0	−2.35	−1.58
500	−1.81	−1.54
1000	−1.25	−1.10
1500	−0.52	−0.47
1685	−0.43	−0.39
1816	0.22	0.16
2000	0.17	0.05
2500	0.71	0.58
3000	1.43	1.12
3132	1.57	1.22

## Data Availability

Data are contained within the article.
